# Charge Storage Properties of Ferrimagnetic BaFe_12_O_19_ and Polypyrrole–BaFe_12_O_19_ Composites

**DOI:** 10.3390/molecules29091979

**Published:** 2024-04-25

**Authors:** Silin Chen, Igor Zhitomirsky

**Affiliations:** Department of Materials Science and Engineering, McMaster University, Hamilton, ON L8S 4L7, Canada; chens411@mcmaster.ca

**Keywords:** barium ferrite, polypyrrole, composite, supercapacitor

## Abstract

This investigation is motivated by an interest in multiferroic BaFe_12_O_19_ (BFO), which combines advanced ferrimagnetic and ferroelectric properties at room temperature and exhibits interesting magnetoelectric phenomena. The ferroelectric charge storage properties of BFO are limited due to high coercivity, low dielectric constant, and high dielectric losses. We report the pseudocapacitive behavior of BFO, which allows superior charge storage compared to the ferroelectric charge storage mechanism. The BFO electrodes show a remarkably high capacitance of 1.34 F cm^−2^ in a neutral Na_2_SO_4_ electrolyte. The charging mechanism is discussed. The capacitive behavior is linked to the beneficial effect of high-energy ball milling (HEBM) and the use of an efficient dispersant, which facilitates charge transfer. Another approach is based on the use of conductive polypyrrole (PPy) for the fabrication of PPy-BFO composites. The choice of new polyaromatic dopants with a high charge-to-mass ratio plays a crucial role in achieving a high capacitance of 4.66 F cm^−2^ for pure PPy electrodes. The composite PPy-BFO (50/50) electrodes show a capacitance of 3.39 F cm^−2^, low impedance, reduced charge transfer resistance, enhanced capacitance retention at fast charging rates, and good cyclic stability due to the beneficial effect of advanced dopants, HEBM, and synergy of the contribution of PPy and BFO.

## 1. Introduction

BaFe_12_O_19_ (BFO) is an advanced ferrimagnetic material for applications in magnetic recording media, microwave devices, sensors, transducers, and electromagnetic shielding [1]. Moreover, the magnetic properties of BFO were used to develop absorbents for the removal of various toxins from water [2]. BFO exhibits high magnetization and magnetic anisotropy, a high Curie temperature of 723 K, and a good maximum energy product value (BH_max_) [3]. BFO shows superior magnetic hardness compared to other hexaferrites. Moreover, BFO has high chemical stability, electrical resistivity, and corrosion resistance [1,4]. The crystallographic unit cell of BFO contains 24 Fe^3+^ ions in three different crystallographic positions. The superexchange interactions result in a magnetic moment of 40 μ_B_ per unit cell. The high remanent magnetization and high coercivity of BFO were beneficial for the fabrication of hard magnetic composites containing BFO particles in a polymer matrix [5,6].

There is currently a surge of interest in the electrical properties of BFO. The deformation of FeO_6_ octahedrons in the BFO magnetoplumbite structure and the off-center shift of Fe^3+^ ions results in polarization, which can be enhanced by doping [3]. Pure and doped BFO showed multiferroic properties [7]. The spontaneous polarization of pure BFO was found [8] to be 48.0–49.5 μC cm^−2^. BFO showed [9] a decrease of the dielectric constant with increasing magnetic field and a magnetodielectric coupling coefficient of 13% at room temperature [9]. The application of an electric field of 20 kV cm^−1^ to BFO resulted in a remanent magnetization decrease by 5–6% and an increase in the magnetic coercive field by 6–8% [9]. The magnetoelectric coupling in BFO resulted in magnetocapacitance of about 4% [9]. A magnetic field-induced polarization was also reported in this material [10]. BFO showed phase transformation from ferroelectric to antiferroelectric and from antiferroelectric to paraelectric phases at relatively high temperatures [10]. The combination of ferroelectricity and magnetism in materials offers possibilities for various applications based on magnetoelectric effects [11]. Despite of high spontaneous polarization, the applications of BFO ferroelectric properties for capacitors present difficulties due to its high ferroelectric coercive field of 441 kV cm^−1^, low dielectric permittivity at room temperature, and high dielectric losses [10]. However, BFO showed interesting redox properties, which can provide a platform for electrochemical charge storage [12] devices. BFO was investigated as a cathode material for magnesium batteries [12] and an anode material for lithium-ion batteries [13]. BFO is currently under investigation for electrical charge storage applications in supercapacitors. The capacitance of graphene electrodes was increased by adding recycled BFO [14]. Various charge storage mechanisms of BFO were suggested, which involved oxygen defects and Fe^2+^/Fe^3+^/Fe^4+^ redox reactions [15]. The capacitive behavior of BFO was tested in KOH electrolytes, and the electrodes that were obtained showed relatively low capacitance of about 20 F g^−1^ and high resistance [16]. Reduced resistance was reported for composites of BFO with conductive polyaniline polymer [17]. Moreover, the addition of BFO to polyaniline resulted in enhanced capacitance, compared to pure polyaniline in H_2_SO_4_ electrolyte [18].

The high magnetization and pseudocapacitive properties of BFO make it a promising magnetically ordered pseudocapacitive (MOPC) material. It is in this regard that many MOPC materials [19], such as Fe_3_O_4_, γ-Fe_2_O_3_, CuFe_2_O_4_, NiFe_2_O_4_, and CoFe_2_O_4_, shown by a factor 10^6^ higher capacitance, compared to multiferroics [19,20,21,22,23,24,25]. MOPC exhibits interesting effects related to the coupling of electrochemical charge storage and magnetic properties. It was hypothesized that the use of MOPC materials could address problems related to the development of high active mass supercapacitors with advanced energy-power characteristics for operation at fast charging speeds [19]. The high magnetization and high coercivity of BFO are beneficial for the creation of local magnetic fields in high active mass electrodes containing small BFO particles. In this case, the magnetohydrodynamic effect [19] can facilitate ion access to the electrode surface and improve capacitive properties at fast charging rates. Moreover, MOPC materials offer advantages for magnetic field enhanced energy efficient capacitive deionization devices. However, limited progress was achieved in the application of BFO for supercapacitors.

The goal of this investigation was the development of advanced BFO and BFO-polypyrrole (PPy) electrodes with high active mass loading for energy storage in supercapacitors. PPy is a conductive redox-active polymer that is used for many advanced energy storage and catalytic applications [26,27,28,29,30,31]. This polymer and its composites exhibit good charge storage properties, which are especially important for supercapacitors [31,32]. The approach developed in this investigation offers the benefits of the use of neutral Na_2_SO_4_ electrolytes. The high capacitance of BFO (1.34 F cm^−2^) was achieved using high-energy ball milling (HEMB) and gallocyanine (GCA) as an advanced dispersant. The catechol group of GCA facilitated its adsorption on BFO particles, whereas the electric charge of this molecule allowed electrostatic dispersion. Moreover, the GCA molecule exhibits valuable redox properties, which were used to facilitate charge transfer. New anionic dopants, such as 1,3,(6,7)-naphthalenetrisulfonic acid trisodium salt hydrate (NTS), 1,3,6,8-pyrenetetrasulfonic acid tetrasodium salt hydrate (PTS) were tested for the fabrication of PPy-NTS and PPy-PTS electrodes with high capacitance of 4.66 F cm^−2^ and the influence of the dopant structure on capacitance was analyzed. The composite electrodes, containing BFO and PPy, showed a capacitance of 3.39 F cm^−2^, good capacitance retention at fast charge/discharge rates, and low impedance due to the synergy of contributions of the individual components.

## 2. Results and Discussion

**Figure 1:**
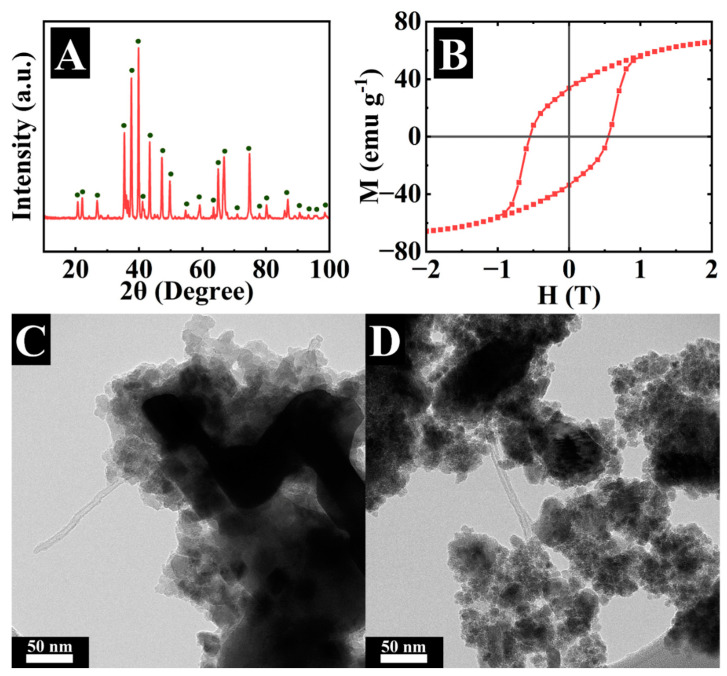
(**A**) X-ray diffraction pattern (●—peaks corresponding to JCPDS file 39-1433), (**B**) magnetization (M) versus magnetic field (H) for as-received BFO, (**C**,**D**) TEM images of (**C**) as-received and (**D**) HEBM BFO.

Figure 1A presents the X-ray diffraction pattern of BFO. The diffraction angles of the peaks and peak intensities are in agreement with JCPDS file 39-1433. Magnetic measurements (Figure 1B) confirmed the hard ferrimagnetic properties of this material, which showed a remanent magnetization of about 50 emu g^−1^ and a coercive field of 0.6 T.

As pointed out above, the use of hard magnetic materials offers advantages for supercapacitor devices for energy storage and magnetically enhanced capacitive water deionization [19]. BFO particles are usually monodomain at particle size below a critical value of about 460 nm [4]. Therefore, the permanent magnetic moment of small BFO particles can facilitate ion transport due to magnetohydrodynamic effects [19]. TEM studies showed that BFO particles were agglomerated due to Van der Waals and magnetic attraction forces. The agglomerates contained large particles with a size of 200–500 nm and smaller particles of about 50 nm (Figure 1C, Supplementary Information, Figure S1). It was hypothesized that HEBM and the use of a dispersant can improve the electrochemical behavior of the electrodes containing BFO. Figure 1D indicates that HEBM resulted in the elimination of large particles with a size above 100 nm. The particle size reduction can be beneficial for fabricating electrodes with enhanced capacitance. However, investigations of different materials did not show a correlation between electrochemical capacitance and BET surface area [33,34,35].

**Figure 2:**
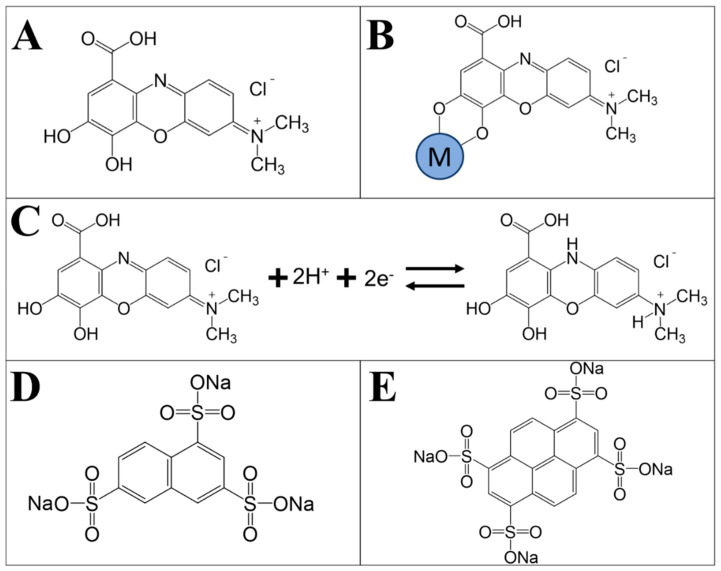
Schematics of (**A**) GCA structure, (**B**) GCA bonding to BFO, involving surface atoms (M = Ba or Fe), (**C**) GCA redox reaction, (**D**) NTS structure and (**E**) PTS structure.

Figure 2A shows a chemical structure of GCA (see experimental section for abbreviations for different materials), which was used as a dispersant for BFO and multiwalled carbon nanotubes (MWCNT, described in Materials and Methods section), which were used as conductive additives. GCA is a polyaromatic cationic molecule containing a catechol group. It was found that GCA allowed enhanced dispersion of BFO and MWCNT in ethanol. Previous investigations [36] showed that as-received MWCNT contained large agglomerates with a typical size of 200–500 μm. The dispersion of MWCNT is critical for their application as conductive additives and utilization of the relatively low percolation limit of MWCNT. Figure 2B illustrates the suggested mechanism of GCA bonding to the Ba or Fe atoms on the BFO surface, which involves a catechol group. This mechanism was reported for other catecholate molecules; it resembles the mechanism of strong bonding of mussel proteins containing catechol groups to inorganic surfaces [37]. The analysis of the literature [38] indicated that non-covalent π-π interactions allowed GCA adsorption on MWCNT. The cationic GCA facilitated the co-dispersion of BFO and MWCNT and their enhanced mixing. Moreover, GCA is a redox-active molecule (Figure 2C), which can act as a charge-transfer mediator for electrochemical reactions [39]. However, due to the low redox charge-to-mass ratio and small amount of GCA used, the direct contribution of this molecule to capacitance is negligibly small. The interest in the application of catecholate molecules as charge-transfer mediators for supercapacitors is motivated by their application as charge-transfer mediators for anodic electropolymerization [40] and photovoltaic devices [41].

**Figure 3:**
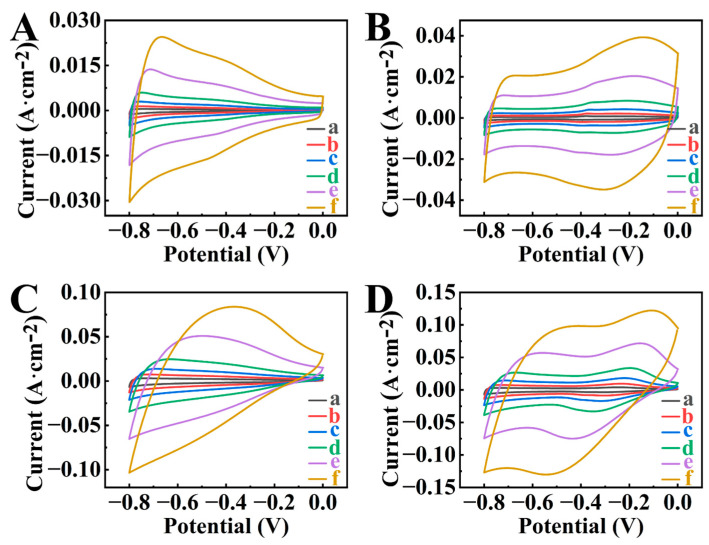
CVs for (**A**) BFO-E, (**B**) BFO-GCA-E, (**C**) HEBMBFO-E, and (**D**) HEBMBFO-GCA-E at sweep rates of (a) 2, (b) 5, (c) 10, (d) 20, (e) 50, and (f) 100 mV s^−1^.

Figure 3 compares cyclic voltammetry (CV) data for different electrodes, such as BFO-E, BFO-GCA-E, HEBMBFO-E, and HEBMBFO-GCA-E. The BFO-E and BFO-GCA-E electrodes contained as-received BFO as an active material. The HEBMBFO-E and HEBMBFO-GCA-E electrodes contained high energy ball milled BFO. GCA was used as a dispersant for as-received BFO and high energy ball milled BFO for preparation of BFO-GCA-E and HEBMBFO-GCA-E electrodes (see also experimental section for abbreviations of different electrodes). The CVs for BFO-E at different sweep rates showed a significant reduction of current with increasing potential from −0.8 to 0 V versus saturated calomel electrode (SCE) (Figure 3A). In contrast, BFO-GCA-E showed enhanced current in the range above −0.4 V and improved CV shape (Figure 3B). A significant increase in current was observed for HEBMBFO-E, which indicated a higher capacitance of such electrodes (Figure 3C). Further increase in current was observed in the investigation of HEBMBFO-GCA-E (Figure 3D). The porous microstructure of such electrodes (Figure S2) was beneficial for electrolyte diffusion and electrolyte access to the active material.

**Figure 4:**
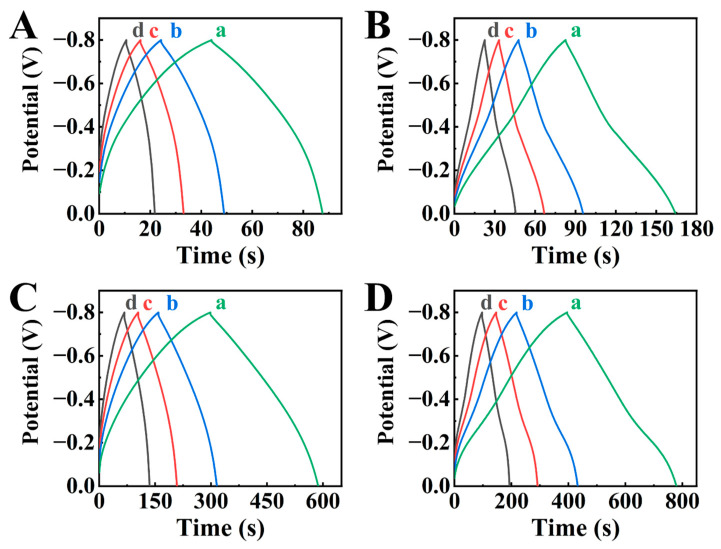
CP data for (**A**) BFO-E, (**B**) BFO-GCA-E, (**C**) HEBMBFO-E, and (**D**) HEBMBFO-GCA-E at current densities of (a) 3, (b) 5, (c) 7, and (d) 10 mA cm^−2^.

**Figure 5:**
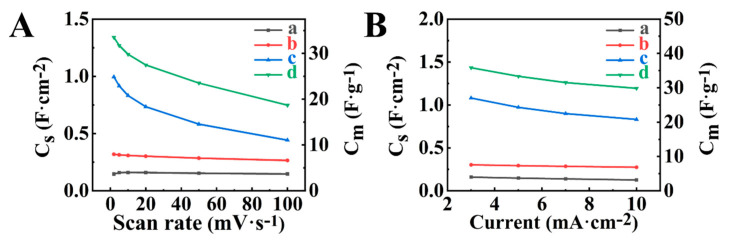
Capacitance (**A**) versus CV sweep rate and (**B**) versus CP current density for (a) BFO-E, (b) BFO-GCA-E, (c) HEBMBFO-E, and (d) HEBMBFO-GCA-E electrodes.

The analysis of chronopotentiometry (CP) data (Figure 4) in the same potential range showed that charge and discharge times increased in an order BFO-E < BFO-GCA-E < HEBMBFO-E < HEBMBFO-GCA-E, which indicated capacitance increase. The shapes of CP curves were close to triangular and symmetric. The capacitances were calculated from CV and CP data and presented in Figure 5 and in Supplementary Information Tables S1 and S2.

The capacitance increased in order BFO-E < BFO-GCA-E < HEBMBFO-E < HEBMBFO-GCA-E for all sweep rates and current densities. Therefore, the testing results show beneficial effects of HEBM and GCA. BMO-E electrodes, prepared using as-received BMO, showed capacitances of 0.15 F cm^−2^ at 2 mV s^−1^ (or 0.16 F cm^−2^ at 3 mA cm^−2^). The use of GCA resulted in capacitances of 0.32 F cm^−2^ at 2 mV s^−1^ (or 0.30 F cm^−2^ at 3 mA cm^−2^) for BFO-GCA-E. HEBM showed a stronger effect on capacitance. The capacitances of HEBMBFO-E and HEBMBFO-GCA-E were found to be 1.00 F cm^−2^ at 2 mV s^−1^ (or 1.08 F cm^−2^ at 3 mA cm^−2^) and 1.34 F cm^−2^ at 2 mV s^−1^ (or 1.43 F cm^−2^ at 3 mA cm^−2^), respectively. The capacitances of HEBMBFO-E and HEBMBFO-GCA-E are comparable with the capacitances of other advanced supercapacitor materials for negative electrodes at similar active mass loadings [42]. For example, nanostructured ferrimagnetic γ-Fe_2_O_3_ showed a capacitance of 1.53 F cm^−2^. As pointed out above, the electrochemical capacitance of supercapacitor materials is superior to the capacitances of ferroelectric and multiferroic materials. Taking into account the literature data [9] for the dielectric permittivity of BFO, the capacitance related to the ferroelectric mechanism is about 10^−11^ F cm^−2^.

The charge storage properties of HEBMBFO-E and HEBMBFO-GCA-E were analyzed using the equation [43]:

*i* = aν^b^ (1)

where *i*—current, ν—CV sweep rate, a and b—parameters. Parameter b = 1 for pure double-layer capacitive response and b = 0.5 for pure battery behavior. Literature data analysis for different materials [44,45,46,47,48] shows that electrodes with 0.5 < b < 1 exhibit a mixed battery and capacitive response. The capacitive behavior is dominant for 0.8 < b < 1. Parameter b was found to be 0.94 and 0.97 for HEBMBFO-E and HEBMBFO-GCA-E electrodes, indicating their pseudocapacitive response. Considering the advanced magnetic properties of BFO and its pseudocapacitive properties, this material can be considered a MOPC [19] material.

The charge storage mechanism of BFO can be described by the following equation:

BaFe^3+^_12_O_19_ + δe^−^ + δNa^+^ ↔ BaFe^3+^_12−δ_ Fe^2+^_δ_ O_19_Na_δ_ (2)

**Figure 6:**
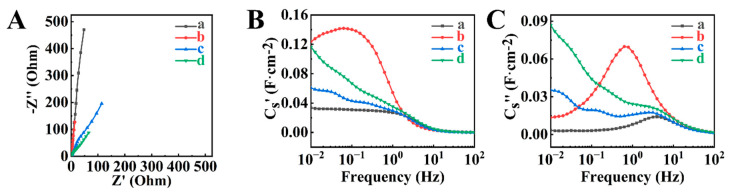
EIS data for (a) BFO-E, (b) BFO-GCA-E, (c) HEBMBFO-E, and (d) HEBMBFO-GCA-E: (**A**) complex impedance presentation in Nyquist plots, (**B**,**C**) components of complex capacitance versus frequency.

Figure 6 and Table S3 show the results of the EIS study of the BFO-E, BFO-GCA-E, HEBMBFO-E, and HEBMBFO-GCA-E electrodes. The use of GCA resulted in lower resistance. BFO-GCA-E showed lower real parts of impedance compared to BFO-E at the same frequencies, and HEBMBFO-GCA-E electrodes showed lower real parts of impedance compared to HEBMBFO-E. The lower real part of impedance indicated lower resistance, which is beneficial for energy storage in supercapacitors. It is in agreement with the literature data, which indicates that GCA can act as a charge-transfer mediator for electrochemical reactions [39] and facilitate charge transfer. However, HEBM resulted in higher resistance. HEBMBFO-E showed higher resistances compared to BMO-E at the same frequencies, whereas HEBMBFO-GCA-E showed higher resistances compared to BFO-GCA-E.

BFO-GCA-E showed the highest AC capacitance at frequencies below 1 Hz and relatively high relaxation frequency, corresponding to the maximum of the imaginary component at ~0.6 Hz. HEBMBFO-GCA-E electrodes showed higher capacitance compared to BFO-E and HEBMBFO-E. The problem of higher resistance of HEBM BFO was addressed by the use of composites with conductive PPy polymer.

**Figure 7:**
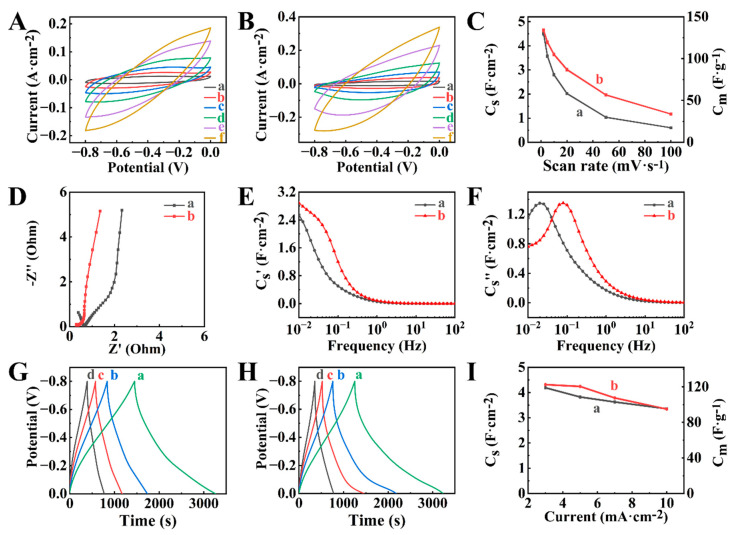
(**A**,**B**) CVs at sweep rates of (a) 2, (b) 5, (c) 10, (d) 20, (e) 50, and (f) 100 mV s^−1^, (**C**) capacitance calculated from CVs versus sweep rate, (**D**–**F**) EIS data, (**G**,**H**) CP data at current densities of (a) 3, (b) 5, (c) 7, and (d) 10 mA cm^−2^ and (**I**) capacitance calculated from CP data versus current density for (A), (C(a)), (D(a)), (E(a)), (F(a)), (G), I(a) PPy-NTS-E and (B), (C(b)), (D(b)), (E(b)), (F(b)). (H), I(b) PPy-PTS-E.

PPy electrodes were prepared using new anionic polyaromatic dopants, such as NTS and PTS. Previous investigations [49] highlighted the benefits of polyaromatic dopants for the fabrication of PPy with enhanced conductivity. Figure 2D,E show the chemical structures of the polyaromatic dopants selected for this study. The chemical structure of NTS and PTS contain 3 and 4 anionic SO_3_^−^ groups, respectively, bonded to the carbon atoms of the aromatic rings. It is known that multiple anionic groups of the dopants can potentially be involved in the doping of different polymer chains [50], enhancing the interchain mobility of charge carriers and improving the electrical conductivity of PPy. Another important parameter for the selection of dopants for PPy application in supercapacitors is a charge-to-mass ratio [51], which is higher for NTS compared to PTS. Figure 7 shows electrochemical testing results for PPy-NTS-E and PPy-PTS-E electrodes, prepared using NTS and PTS dopants, respectively.

**Figure 8:**
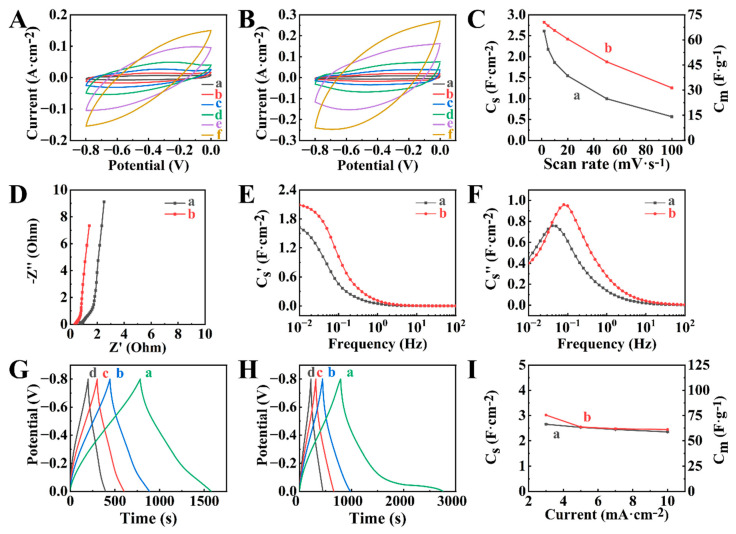
(**A**,**B**) CVs at sweep rates of (a) 2, (b) 5, (c) 10, (d) 20, (e) 50, and (f) 100 mV s^−1^, (**C**) capacitance calculated from CVs versus sweep rate, (**D**–**F**) EIS data, (**G**,**H**) CP data at current densities of (a) 3, (b) 5, (c) 7, and (d) 10 mA cm^−2^ and (**I**) capacitance calculated from CP data versus current density for (A), (C(a)), (D(a)), (E(a)), (F(a)), (G), I(a) BFO-PPy-NTS-E and (B), (C(b)), (D(b)), (E(b)), (F(b)), (H), I(b) BFO-PPy-PTS-E.

The electrochemical studies did not show redox peaks in the CVs (Figure 7A,B). The capacitances at 2 mV s^−1^ were found to be 4.53 and 4.66 F cm^−2^ for PPy-NTS-E and PPy-PTS-E. PPy-PTS-E showed better capacitance retention at high sweep rates (Figure 7C). EIS data showed lower resistance of PPy-PTS-E compared to PPy-NTS-E (Figure 7D). Moreover, PPy-PTS-E showed higher real components of complex capacitance (Figure 7E). The relaxation frequencies were found to be 0.02 and 0.08 Hz for PPy-NTS-E and PPy-PTS-E, respectively (Figure 7F). The analysis of CP data (Figure 7G,H) showed comparable charge-discharge times for PPy-NTS-E and PPy-PTS-E. The capacitances of 4.18 and 4.30 F cm^−2^ were obtained (Figure 7I) for PPy-NTS-E and PPy-PTS-E, respectively, at 3 mA cm^−2^. Figure 8 shows electrochemical testing results for composite BFO-PPy-NTS-E and BFO-PPy-PTS-E electrodes, prepared using as-received BFO and PPy, doped with NTS and PTS, respectively.

The CVs for BFO-PPy-NTS-E and BFO-PPy-PTS-E did not show redox peaks (Figure 8A,B). The capacitances of 2.61 and 2.82 F cm^−2^ were obtained for BFO-PPy-NTS-E and BFO-PPy-PTS-E, respectively, at 2 mV s^−1^. BFO-PPy-PTS-E showed improved capacitance retention (Figure 8C) in the sweep rate range 2–100 mV s^−1^, compared to BFO-PPy-NTS-E. The analysis of EIS data showed relatively low electrode resistance, which was comparable with the resistances of PPy-NTS-E and PPy-PTS-E polymer-based electrodes and lower than the resistance of BFO-E (Table S3). The investigations of frequency dependences of complex capacitance (Figure 8E,F) showed a higher real part of the capacitance of BFO-PPy-PTS-E and higher relaxation frequency compared to BFO-PPy-NTS-E (Table S3). CP studies of the charge-discharge behavior of the electrodes (Figure 8G–I) showed capacitances of 2.65 and 3.02 F cm^−2^ for BFO-PPy-NTS-E and BFO-PPy-PTS-E, respectively.

**Figure 9:**
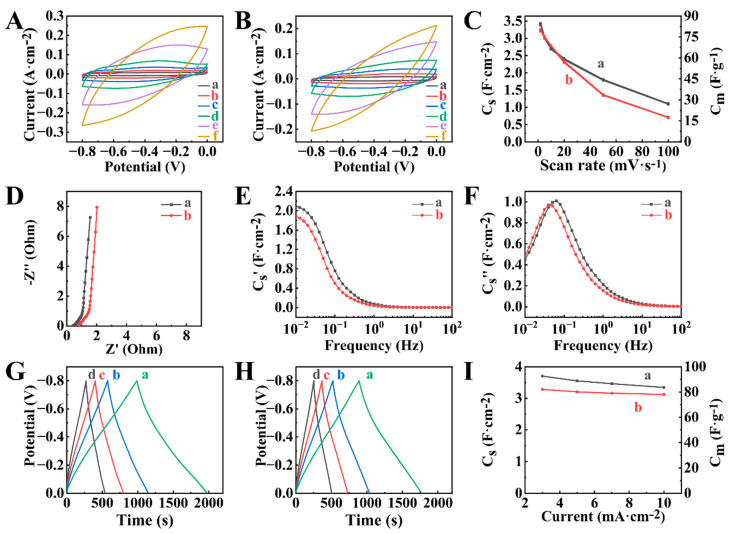
(**A**,**B**) CVs at sweep rates of (a) 2, (b) 5, (c) 10, (d) 20, (e) 50, and (f) 100 mV s^−1^, (**C**) capacitance calculated from CVs versus sweep rate, (**D**–**F**) EIS data, (**G**,**H**) CP data at current densities of (a) 3, (b) 5, (c) 7, and (d) 10 mA cm^−2^ and (**I**) capacitance calculated from CP data versus current density for (A), (C(a)), (D(a)), (E(a)), (F(a)), (G), I(a) HEBMBFO-PPy-NTS-E and (B), (C(b)), (D(b)), (E(b)), (F(b)). (H), I(b) HEBMBFO-PPy-PTS-E.

The use of HEBM BFO resulted in higher capacitances of the composite electrodes compared to electrodes containing as-received BFO. The CVs for the composite (Figure 9A,B) electrodes did not show redox peaks. The capacitances (Figure 9C) of 3.39 and 3.21 F cm^−2^ were obtained for HEBMBFO-PPy-NTS-E and HEBMBFO-PPy-PTS-E, prepared using high energy ball milled BFO and PPy, doped with NTS and PTS, respectively, at 2 mV s^−1^. The obtained capacitances were higher compared to the capacitances of BFO-PPy-NTS-E and BFO-PPy-PTS-E electrodes prepared with as-received BFO. The electrodes showed a significant increase in capacitance and reduction in resistance (Figure 9D) compared to HEBMBFO-E. Therefore, the use of PPy facilitated the fabrication of magnetic composites with high capacitance and low resistance. The investigations of the real part of complex capacitance (Figure 9E) confirmed improved capacitive behavior of HEBMBFO-PPy-NTS-E and HEBMBFO-PPy-PTS-E, compared to HEBMBFO-E. The relaxation frequencies for HEBMBFO-PPy-NTS-E and HEBMBFO-PPy-PTS-E were found to be 63 and 49 mHz, respectively (Figure 9F). The enhanced capacitance of the HEBMBFO-PPy-NTS-E and HEBMBFO-PPy-PTS-E composites is also evident from the analysis of the CP data at different current densities (Figure 9G–I), showing longer charge-discharge times, which resulted in capacitances of 3.70 and 3.28 F cm^−2^ for HEBMBFO-PPy-NTS-E and HEBMBFO-PPy-PTS-E, respectively at 3 mA cm^−2^.

The analysis of electrochemical testing data using Equation (1) showed that parameters b were 0.89 and 0.79 for HEBMBFO-PPy-NTS-E and HEBMBFO-PPy-PTS-E, respectively, which indicated pseudocapacitive response of the electrodes. EIS data was also analyzed using an equivalent circuit presented in Figure S3 (Supplementary Information), which was developed for electrodes with high active mass loadings [52]. It is known that equivalent circuits of porous supercapacitor electrodes are usually described by a transmission line containing multiple (typically 5–10) RC elements [53,54,55,56]. The equivalent circuit used in our investigation also contained a transmission line with only 2 RC/RQ elements (Q-constant phase capacitive element). The simulation data was in agreement with experimental data (Figures S4 and S5). The electrodes showed charge transfer resistances of 0.10 and 0.13 Ohm for HEBMBFO-PPy-NTS-E and HEBMBFO-PPy-PTS-E, respectively. The analysis of SEM images (Figure S2) of HEBMBFO-PPy-NTS-E and HEBMBFO-PPy-PTS-E showed their porous microstructure, which facilitated electrolyte access to the active material.

**Figure 10:**
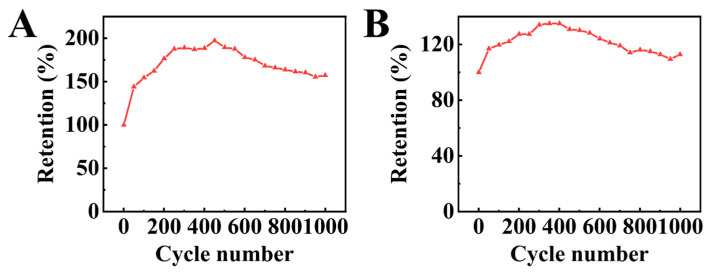
Capacitance retention for (**A**) HEBMBFO-PPy-NTS-E and (**B**) HEBMBFO-PPy-PTS-E.

The investigation of cyclic stability of the composite electrodes showed an initial increase in capacitance (Figure 10) during cycling. The capacitance retention after 1000 cycles was found to be 157 and 113% for HEBMBFO-PPy-NTS-E and HEBMBFO-PPy-PTS-E, respectively. The capacitance increase can result from different factors, such as changes in the bulk microstructure of the electrodes, electrode material activation, and enhanced wetting [57].

Experiments were also performed for a better illustration of the beneficial effects of GCA as a chelating dispersing agent for HEBMBFO and NTS and PTS as dopants for PPy. The HEBMBFO-NTS-E and HEBMBFO-PTS-E, prepared using NTS and PTS, respectively, as dispersants, did not show any improvement in capacitance compared to HEBMBFO-E (Figure S6). The CV, EIS, and CP data showed significantly lower capacitance of HEBMBFO-NTS-E and HEBMBFO-PTS-E electrodes compared to the capacitance of HEBMBFO-GCA-E. Therefore, the catechol ligand of GCA is critically important for the adsorption and dispersion of this molecule on HEBMBFO. The HEBMBFO-PPy-GCA-E prepared using GCA as a dopant for PPy, showed lower capacitance compared to the HEBMBFO-PPy-NTS-E and HEBMBFO-PPy-PTS-E electrodes. The corresponding CV, EIS, and CP data (Figure S7) provide evidence of the beneficial effect of NTS and PTS as dopants.

## 3. Materials and Methods

### 3.1. Materials

Pyrrole (Py), BaFe_12_O_19_, gallocyanine (GCA), 1,3,(6,7)-naphthalenetrisulfonic acid trisodium salt hydrate (NTS), 1,3,6,8-pyrenetetrasulfonic acid tetrasodium salt hydrate (PTS), ammonium persulfate (APS) and polyvinyl butyral (PVB) were purchased from Millipore Sigma (Mississauga, ON, Canada). MWCNT (diameter 13 nm, length 1–2 μm) was provided by Bayer (Leverkusen, Germany). Ni foam current collectors with 95% volumetric porosity were supplied by Vale (Mississauga, ON, Canada). Py was stored in a fridge at 4 °C before use. All chemicals were used as received without further purification. BFO was a high-energy ball milled using a Mixer Mill MM 500 Nano (Retsch GmbH, Haan, Germany) for 2 h at a frequency of 15 Hz. After the milling process, the material was washed with ethanol and dried.

### 3.2. Synthesis of PPy

PPy was prepared by a chemical polymerization reaction with APS as the oxidant at 4 °C. Synthesis of PPy, doped with NTS (PPy-NTS) or PTS (PPy-PTS) involved preparation of 100 mL of 1 mM NTS or 1 mM PTS solutions, containing 10 mM Py in DI water at 4 °C, stirring for 10 min, and slowly adding 50 mL of 0.2 M APS solution in DI water. The resultant mixture was subsequently allowed to react during 2 h at 4 °C. The precipitated PPy materials were filtered and washed with 1 L DI water. The obtained powders were dried at 60 °C for 12 h.

### 3.3. Electrode Fabrication

All electrodes tested in this investigation contained active materials (AM) and MWCNT in a mass ratio AM:MWCNT = 4:1. Various AM, including as-received BFO, HEBM BFO, PPy-NTS, PPy-PTS and composite powders of BFO and PPy-NTS, BFO and PPy-PTS, HEBM BFO and PPy-NTS, HEBM BFO and PPy-PTS were mixed with MWCNT in ethanol, containing dissolved PVB for the preparation of slurries for impregnation of current collectors and fabrication of electrodes BFO-E, HEBMBFO-E, PPy-NTS-E, PPy-PTS-E, BFO-PPy-NTS-E, BFO-PPy-PTS-E, HEBMBFO-PPy-NTS-E, HEBMBFO-PPy-PTS-E, respectively. The mass ratio of BFO or HEBM BFO to PPy-NTS or PPy-PTS was 1:1.

In another approach, BFO or HEBM BFO were mixed with MWCNT in ethanol containing GCA dispersant and PVB for the preparation of slurries for impregnation of current collectors and fabrication of electrodes BFO-GCA-E and HEBMBFO-GCA-E. The mass ratio of GCA: (AM + MWCNT) was 0.02. The mass ratio of PVB to (AM + MWCNT) was 0.03 all the slurries.

The slurries were ultrasonically agitated before the impregnation of current collectors. After drying, the impregnated current collectors with initial thickness of 1.6 mm were pressed to a final thickness of 0.5 mm. The mass of the impregnated material was limited to 0.035 g cm^−2^ for PPy-NTS-E and PPy-PTS-E due to low density of PPy. For other electrodes of the same thickness the mass of the impregnated material was 0.040 g cm^−2^.

### 3.4. Characterization Methods

XRD studies were conducted using a Bruker D8 Advance diffractometer with Cu-Kα radiation. TALOS L102C microscope (Thermo Fisher Scientific, Waltham, MA, USA), was used for transmission electron microscopy (TEM) investigations. Magnetic investigations were carried out with a Quantum Design SQUID magnetometer (San Diego, CA, USA). The electrochemical testing cell for chronopotentiometry (CP), cyclic voltammetry (CV), and AC electrochemical impedance spectroscopy (EIS) studies contained a saturated calomel electrode (SCE) as a reference, Pt mesh counter electrode, and a working electrode in 0.5 M Na_2_SO_4_ electrolyte. EIS studies were performed at an amplitude of AC signal of 5 mV. The equations used for the capacitance calculations from the CP, CV, and EIS data with SP300 Biologic potentiostat were previously described [42].

Areal (C_S_) and gravimetric (C_m_) capacitances were derived from the CV data using the following equation:

(3) $$C=\frac{\Delta Q}{\Delta U}=\frac{|\int_{0}^{t(U_{max})}Idt|+|\int_{t(U_{max})}^{0}Idt|}{2U_{max}}$$

where Δ*Q* is charge, *I* is current, and Δ*U* is the potential range, and from the chronopotentiometry data:

C = IΔt/ΔU(4)

The complex capacitance C*(ω) = C′(ω) − *i*C″(ω) was derived at different frequencies (ω) from the complex impedance Z*(ω) = Z′(ω) + *i* Z″(ω):

(5) $$C^{\prime }\left(\omega \right)=\frac{-Z^{\prime \prime }(\omega )}{\omega {\left|Z(\omega )\right|}^{2}}$$

(6) $$C^{\prime }\prime \left(\omega \right)=\frac{Z^{\prime }(\omega )}{\omega {\left|Z(\omega )\right|}^{2}}$$

## 4. Conclusions

Multiferroic BFO exhibited advanced pseudocapacitive properties in Na_2_SO_4_ electrolytes. The pseudocapacitive charge storage mechanism allowed superior charge storage properties compared to the ferroelectric charge storage mechanism. The use of HEBM and GCA allowed better utilization of the pseudocapacitive properties of BFO. The HEBMBFO-E and HEBMBFO-GCA-E electrodes showed capacitances of 1.00 F cm^−2^ and 1.34 F cm^−2^ at 2 mV s^−1^, respectively. The pseudocapacitive charge storage mechanism is attributed to the reduction of Fe^3+^ ions. New anionic polyaromatic dopants with a high charge-to-mass ratio were tested for the fabrication of PPy electrodes. The capacitances at 2 mV s^−1^ were found to be 4.53 and 4.66 F cm^−2^ for PPy-NTS-E and PPy-PTS-E. The composite HEBMBFO-PPy-NTS-E and HEBMBFO-PPy-PTS-E electrodes showed capacitances of 3.39 and 3.21 F cm^−2^, respectively, at 2 mV s^−1^. Moreover, the high active mass composite electrodes showed low impedance, reduced charge transfer resistance enhanced capacitance retention at fast charging rates, and good cyclic stability due to beneficial effect of the advanced dopants, HEBM and synergy of contribution of PPy and BFO. The BFO and BFO-PPy electrodes combine advanced pseudocapacitive and magnetic properties and can be used for anodes of magnetic supercapacitors.

## Data Availability

The data is available in this manuscript, as well as Supplementary Information.

## Supplementary Materials

The following supporting information can be downloaded at: https://www.mdpi.com/article/10.3390/molecules29091979/s1; Figure S1: TEM image of as-received BFO; Figure S2: SEM images of electrodes; Figure S3: Equivalent circuit used for EIS data simulation [52]; Figures S4 and S5: EIS simulation data, Figures S6 and S7: CV, EIS, CP data. Table S1: Capacitances, calculated from CV data; Table S2: Capacitance calculated from CP data; Table S3: EIS data for different electrodes.
