# Supplementary material for: Charge Storage Properties of Ferrimagnetic BaFe12O19 and Polypyrrole–BaFe12O19 Composites
Source: Molecules. 2024 Apr 25;29(9):1979. doi: 10.3390/molecules29091979 (PMC11085603; doi:10.3390/molecules29091979)
Supplement: Supplementary file 1 [file molecules-29-01979-s001.zip › molecules-2880852-supplementary.pdf]

Supplementary Information

Charge storage properties of ferromagnetic $BaFe_{12}O_{19}$ and polypyrrole – $BaFe_{12}O_{19}$ composites

Silin Chen and Igor Zhitomirsky*

Department of Materials Science and Engineering, McMaster University,

1280 Main Street West, L8S 4L8, Hamilton, Ontario Canada

*Email: zhitom@mcmaster.ca

Table S1. Capacitances, calculated from CV data.

Electrode	Capacitances C_s (F cm^{-2}) / C_m(F g^{-1}) at different sweep rates (mVs^{-1})					
	**2 mVs^{-1}**<br>C_s (Fcm^{-2})<br>C_m (Fg^{-1})	**5 mVs^{-1}**<br>C_s (Fcm^{-2})<br>C_m (Fg^{-1})	**10 mVs^{-1}**<br>C_s (Fcm^{-2})<br>C_m (Fg^{-1})	**20 mVs^{-1}**<br>C_s (Fcm^{-2})<br>C_m (Fg^{-1})	**50 mVs^{-1}**<br>C_s (Fcm^{-2})<br>C_m (Fg^{-1})	**100 mVs^{-1}**<br>C_s (Fcm^{-2})<br>C_m (Fg^{-1})
BFO-E	0.146<br>3.650	0.159<br>3.963	0.160<br>3.988	0.159<br>3.974	0.153<br>3.823	0.147<br>3.670
HEBMBFO-E	1.00<br>24.88	0.91<br>22.90	0.83<br>20.80	0.73<br>18.35	0.58<br>14.55	0.44<br>11.05
BFO-GCA-E	0.319<br>7.97	0.313<br>7.84	0.308<br>7.71	0.301<br>7.54	0.285<br>7.12	0.265<br>6.63
HEBMBFO-GCA-E	1.34<br>33.51	1.27<br>31.73	1.19<br>29.82	1.10<br>27.49	0.94<br>23.52	0.75<br>18.69
PPy-NTS-E	4.53<br>129.38	3.59<br>102.50	2.81<br>80.36	2.03<br>58.00	1.04<br>29.75	0.61<br>17.29
PPy-PTS-E	4.66<br>133.26	4.17<br>119.03	3.66<br>104.48	3.03<br>86.47	1.97<br>56.32	1.18<br>33.65
BFO-PPy-NTS-E	2.61<br>65.19	2.17<br>54.37	1.86<br>46.47	1.54<br>38.54	0.99<br>24.86	0.57<br>14.16
BFO-PPy-PTS-E	2.82<br>70.45	2.74<br>68.43	2.62<br>65.62	2.42<br>60.52	1.88<br>46.93	1.26<br>31.38
HEBMBFO-PPy-NTS-E	3.39<br>84.71	3.00<br>75.03	2.68<br>67.11	2.38<br>59.48	1.78<br>44.60	1.09<br>27.27
HEBMBFO-PPy-PTS-E	3.21<br>80.26	3.01<br>75.36	2.75<br>68.76	2.30<br>57.55	1.35<br>33.72	0.70<br>17.55

Table S2. Capacitance calculated from CP data.

Electrode	Capacitances C_s (F cm^{-2})/C_m(F g^{-1}) at different current density (mAcm^{-2})			
	**3 mAcm^{-2}**<br>C_s (Fcm^{-2})<br>C_m (Fg^{-1})	**5 mAcm^{-2}**<br>C_s (Fcm^{-2})<br>C_m (Fg^{-1})	**7 mAcm^{-2}**<br>C_s (Fcm^{-2})<br>C_m (Fg^{-1})	**10 mAcm^{-2}**<br>C_s (Fcm^{-2})<br>C_m (Fg^{-1})
BFO-E	0.16<br>3.99	0.15<br>3.70	0.14<br>3.48	0.13<br>3.18
HEBMBFO-E	1.08<br>27.03	0.97<br>24.31	0.90<br>22.50	0.83<br>20.78
BFO-GCA-E	0.30<br>7.57	0.29<br>7.33	0.28<br>7.14	0.27<br>6.87
HEBMBFO-GCA-E	1.43<br>35.85	1.33<br>33.34	1.26<br>31.52	1.19<br>29.83
PPy-NTS-E	4.18<br>119.35	3.81<br>108.88	3.61<br>103.24	3.35<br>95.60
PPy-PTS-E	4.30<br>122.87	4.23<br>120.83	3.77<br>107.83	3.33<br>95.17
BFO-PPy-NTS-E	2.65<br>66.37	2.53<br>63.30	2.45<br>61.21	2.35<br>58.79
BFO-PPy-PTS-E	3.02<br>75.50	2.55<br>63.65	2.48<br>61.93	2.44<br>61.12
HEBMBFO-PPy-NTS-E	3.70<br>92.56	3.55<br>88.85	3.46<br>86.60	3.35<br>83.65
HEBMBFO-PPy-PTS-E	3.28<br>82.08	3.20<br>80.10	3.16<br>79.04	3.12<br>78.07

Table S3. EIS data for different electrodes

Electrode	Z' at 10 mHz<br>Ohm	C' at 10 mHz<br>F cm^{-2}
BFO-E	47.90	0.033
HEBMBFO-E	113.95	0.061
BFO-GCA-E	13.86	0.124
HEBMBFO-GCA-E	65.41	0.117
PPy-NTS-E	2.33	2.55
PPy-PTS-E	1.36	2.89
BFO-PPy-NTS-E	2.51	1.62
BFO-PPy-PTS-E	1.40	2.08
HEBMBFO-PPy-NTS-E	1.56	2.09
HEBMBFO-PPy-PTS-E	2.01	1.88

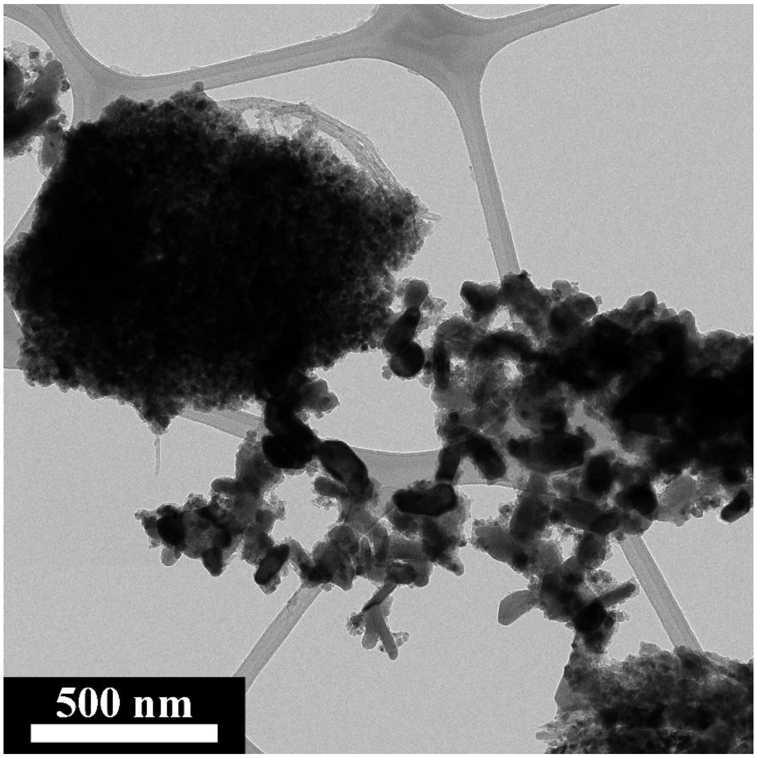

Figure S1. TEM image of as-received BFO obtained using TALOS L102C microscope (Thermo Fisher Scientific)

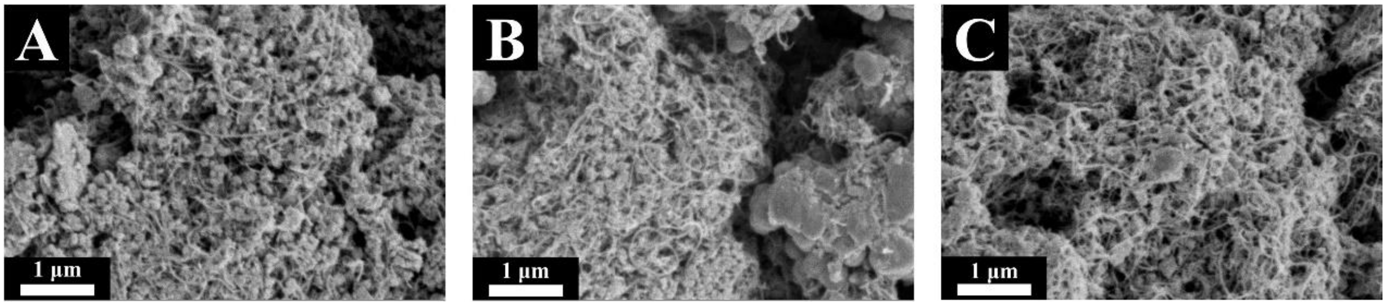

Figure S2. SEM images of (A) HEBMBFO-GCA-E, (B) HEBMBFO-PPy-NTS-E, and (C) HEBMBFO-PPy-PTS-E, obtained using Apreo 2 S LoVac microscope microscope (Thermo Fisher Scientific)

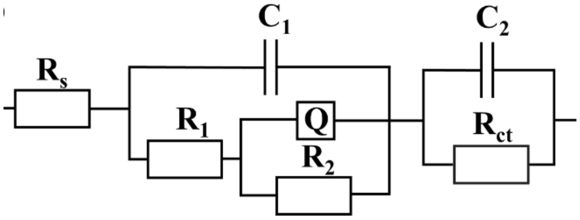

Figure S3.Equivalent circuit used for EIS data simulation[1]

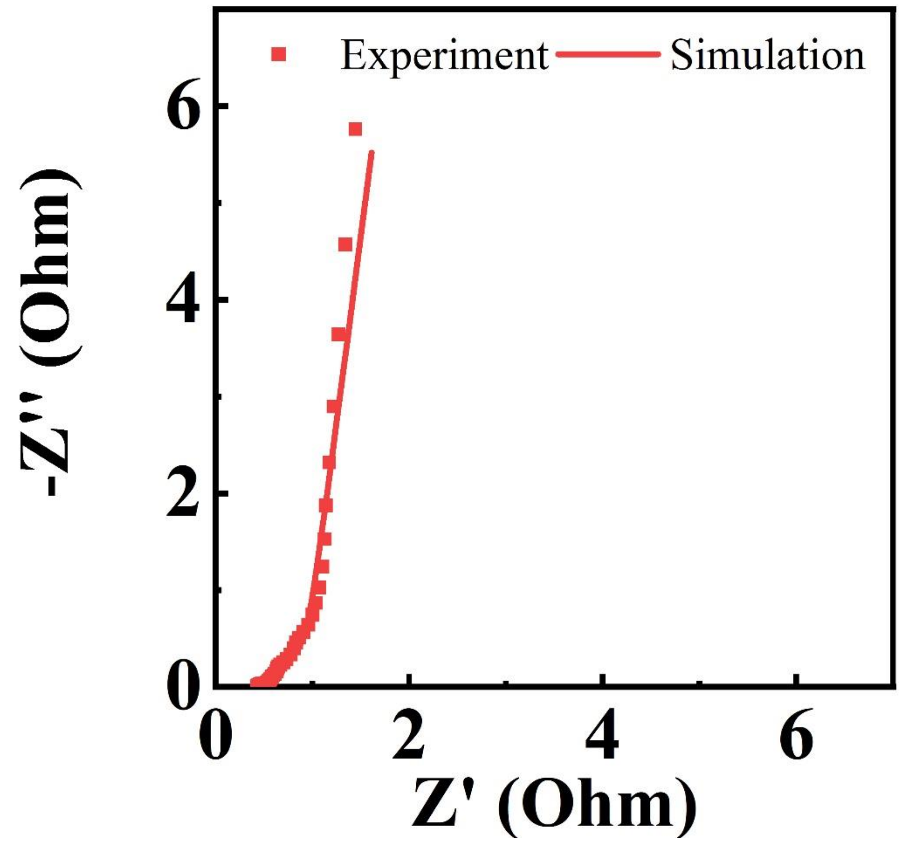

Figure S4. EIS data for HEBMBFO-PPy-NTS-E.

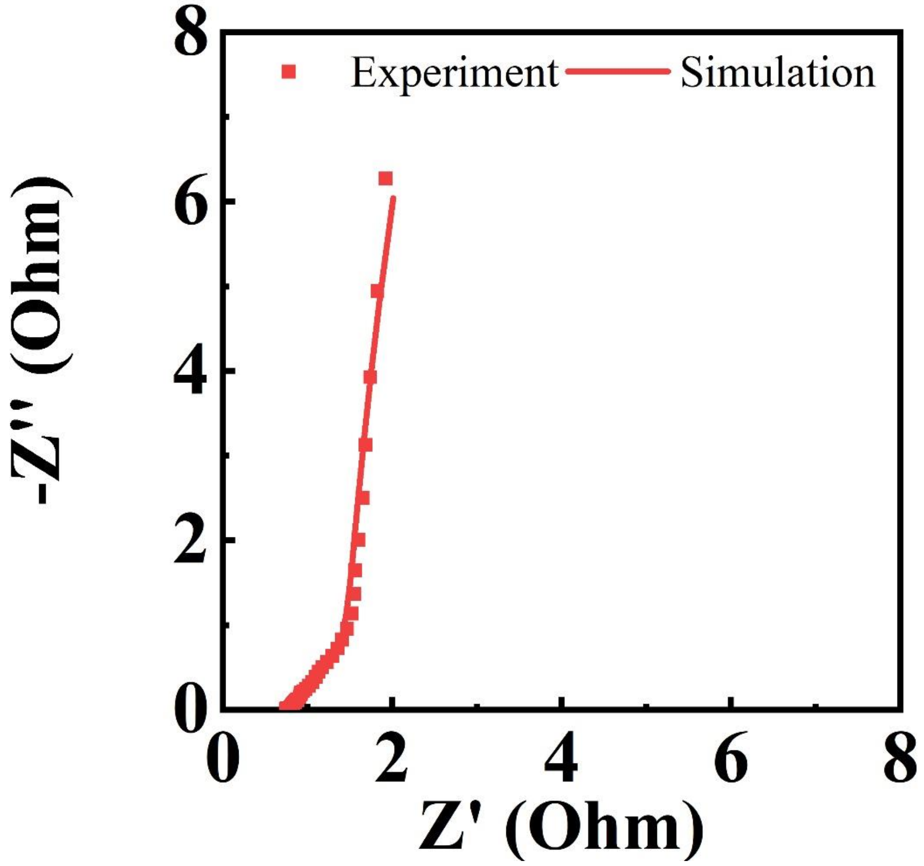

Figure S5. EIS data for HEBMBFO-PPy-PTS-E

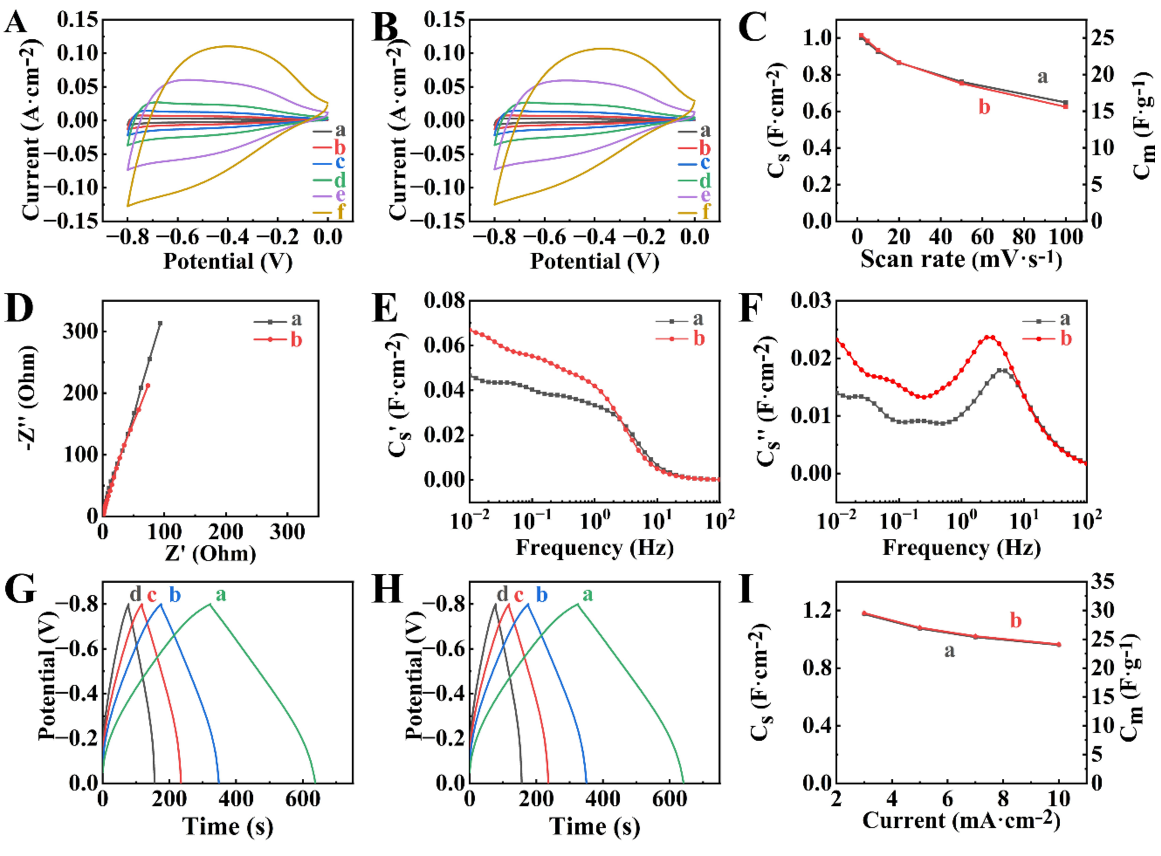

Figure S6. (A, B) CVs at sweep rates of (a) 2, (b) 5, (c) 10, (d) 20, (e) 50, and (f) 100 mV s^{-1}, (C) capacitance calculated from CVs versus sweep rate, (D-F) EIS data, (G, H) CP data at current densities of (a) 3, (b) 5, (c) 7, and (d) 10 mA cm^{-2} and (I) capacitance calculated from CP data versus current density for (A), (C(a)), (D(a)), (E(a)), (F(a)), (G), I(a) HEBM-BFO-NTS-E and (B), (C(b)), (D(b)), (E(b)), (F(b)). (H), I(b) HEBM-BFO-PTS-E.

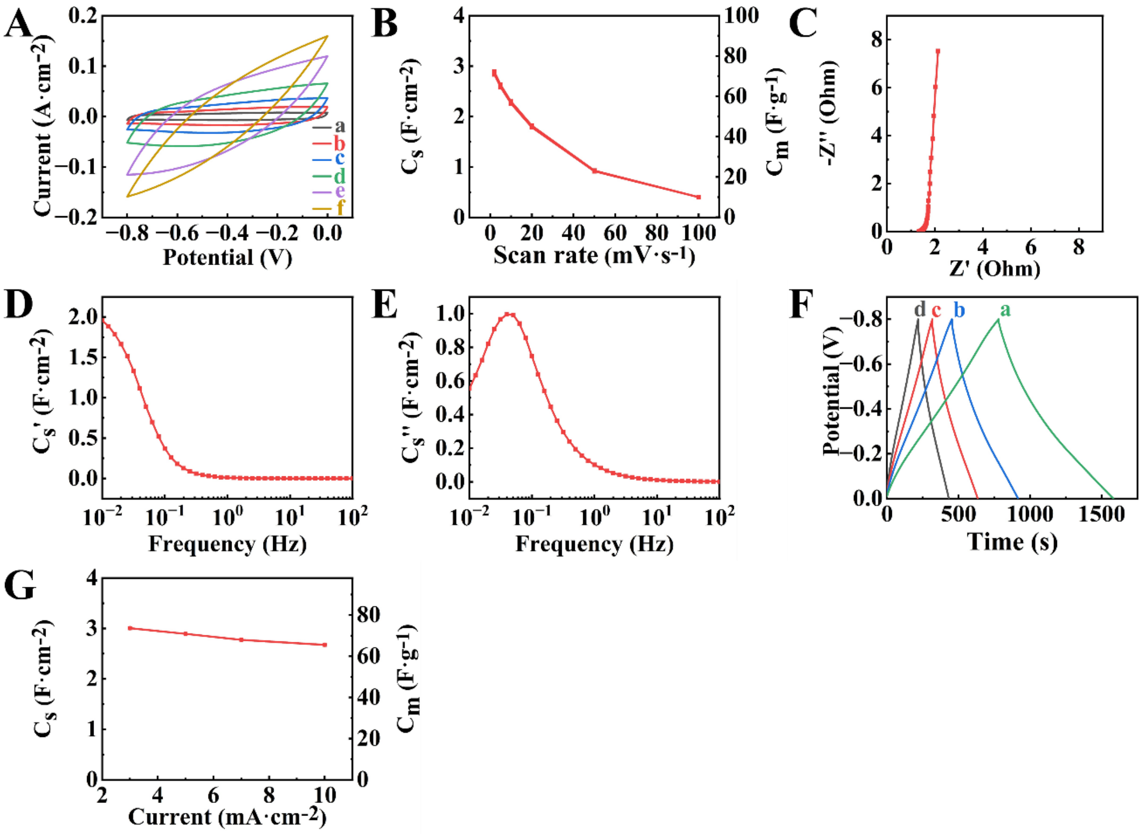

Figure S7 (A) CVs at sweep rates of (a) 2, (b) 5, (c) 10, (d) 20, (e) 50, and (f) 100 mV s^{-1}, (B) capacitance calculated from CVs versus sweep rate, (C-E) EIS data, (F) CP data at current densities of (a) 3, (b) 5, (c) 7, and (d) 10 mA cm^{-2} and (G) capacitance calculated from CP data versus current density for HEBM BFO-PPy-GCA-E.

[1] Y. Wang, Y. Liu, I. Zhitomirsky, Surface modification of MnO 2 and carbon nanotubes using organic dyes for nanotechnology of electrochemical supercapacitors, Journal of Materials Chemistry A 1(40) (2013) 12519-12526.
